# Should involvement in a trial be discussed at a bereavement follow up visit? Views of clinicians and bereaved parents from the bracelet study (bereavement and randomised controlled trials)

**DOI:** 10.1186/1745-6215-14-S1-O81

**Published:** 2013-11-29

**Authors:** Claire Snowdon, Peter Brocklehurst, Robert Tasker, Martin Ward Platt, Diana Elbourne

**Affiliations:** 1 London School of Hygiene and Tropical Medicine, London, UK; 2 National Perinatal Epidemiology Unit, University of Oxford, Oxford, UK; 3 Institute for Women’s Health, University College London, London, UK; 4 Department of Neurology, and Anaesthesia (Pediatrics), Harvard Medical School, Boston, USA; 5 Newcastle Neonatal Service, Royal Victoria Infirmary, Newcastle-upon-Tyne, UK

## 

When patients die, bereaved families may be offered support by their local clinicians. Patients recruited into trials are both patients and trial participants. The BRACELET Study considered whether any response for parents of babies who died after enrolment in a neonatal intensive care (NIC) trial might be triggered by a baby's status as trial participant, at a bereavement follow-up (BFU) appointment.

Thirty five clinicians and 51 bereaved parents involved with five NIC trials were interviewed. Most clinical interviewees stated they would not change their approach to supporting parents in the BFU because a baby had been enrolled in a trial as they believed it was not an issue parents wanted to discuss, it was not relevant, and/or it was not a topic for which relevant clinical information, such as impact of a trial intervention, would be available.

The parents largely confirmed these views. This could be because at the time of the BFU they did not want to discuss trial participation; and/or did not think about discussing trial participation.

The significance of a trial changed over time for the majority of parents. By the time of the interviews, mostly some years after the death, many had become more keen to engage in questions about the research. By this time however, they were usually no longer in contact with either the neonatologists or the trials coordinators.

Further research is needed to consider how best to provide longer term support for families whose relatives died after trial participation.

